# A Low-Power Bio-Potential Acquisition System with Flexible PDMS Dry Electrodes for Portable Ubiquitous Healthcare Applications

**DOI:** 10.3390/s130303077

**Published:** 2013-03-04

**Authors:** Chih-Yuan Chen, Chia-Lin Chang, Chih-Wei Chang, Shin-Chi Lai, Tsung-Fu Chien, Hong-Yi Huang, Jin-Chern Chiou, Ching-Hsing Luo

**Affiliations:** 1 Instrumentation Chip Group, Department of Electric Engineering, National Cheng Kung University, 701 Tainan, Taiwan; E-Mails: n28981442@mail.ncku.edu.tw (C.-Y.C.); c.l.chang1129@gmail.com (C.-L.C.); chingivan2008@gmail.com (S.-C.L.); 2 Department of Bioengineering, University of California, Los Angeles, CA 90095, USA; E-Mail: cw.chang@ucla.edu; 3 Department of Electrical Engineering, Southern Taiwan University, 710 Tainan, Taiwan; E-Mail: jeng12@mail.stust.edu.tw; 4 Graduate Institute of Electrical Engineering, National Taipei University, 237 Taipei, Taiwan; E-Mail: hyhuang@ee.fju.edu.tw; 5 Department of Electrical Engineering, National Chiao Tung University, 300 Hsinchu, Taiwan; E-Mail: chiou@mail.nctu.edu.tw

**Keywords:** dry electrode, PDMS, PMMA, bio-potential acquisition system, ECG

## Abstract

This work describes a bio-potential acquisition system for portable ubiquitous healthcare applications using flexible polydimethylsiloxane dry electrodes (FPDEs) and a low-power recording circuit. This novel FPDE used Au as the skin contact layer, which was made using a CO_2_ laser and replica method technology. The FPDE was revised from a commercial bio-potential electrode with a conductive snap using dry electrodes rather than wet electrodes that proposed reliable and robust attachment for the purpose of measurement, and attaching velcro made it wearable on the forearm for bio-potential applications. Furthermore, this study proposes a recording device to store bio-potential signal data and provides portability and low-power consumption for the proposed acquisition system. To acquire differential bio-potentials, such as electrocardiogram (ECG) signals, the proposed recording device includes a low-power front-end acquisition chip fabricated using a complementary metal-oxide-semiconductor (CMOS) process, a commercial microcontroller (MSP430F149), and a secure digital (SD) card for portable healthcare applications. The proposed system can obtain ECG signals efficiently and are comfortable to the skin. The power consumption of the system is about 85 mW for continuous working over a 3 day period with two AA batteries. It can also be used as a compact Holter ECG system.

## 1. Introducation

A recent surge of research on wireless bio-potential acquisition devices has made healthcare more convenient and comfortable [1–4]. However, more recently, the ubiquitous healthcare data acquisition device literature has paid increasing attention to low-power biomedical chips [5–7]. These studies have resulted in bio-potential recording devices and improvements in patient care. Recording bio-potential acquisition systems usually requires electrodes with several points, an analog-to-digital converter (ADC) that digitizes the amplified signals, and an analog conditioning stage that amplifies and filters the signal, which is followed by a processing unit that manages and processes bio-differential signals such as heart beat rate or QRS complex waves.

Ambulatory bio-potential monitoring is a good method for detecting heart disease and is useful in many situations, including community clinics, in homes and in hospitals. Therefore, several studies have attempted to provide continuous bio-potential monitoring for situations occurring in everyday life. A good example of ECG monitoring is the Holter system [8–11]. The subject can basically record ECG data for 24 hours. However, bio-electrodes (e.g., wet electrodes) have been traditionally used to record ECG signals, and long term application of wet electrodes may result in skin irritation and signal degradation due to dehydration [12]. Therefore, finding comfortable and convenient methods for ECG measurement are very important. Moreover, this work not only provides a low-power acquisition system but also proposes new flexible dry electrodes for the purpose of improving ECG measurement.

Bio-potential electrodes transform the signals from the skin tissue to the acquisition device. Bio-electrodes (e.g., wet electrodes) have traditionally been used to record bio-potential signals by using electric gel to improve electric conductivity. This method is not optimal because its long-term use can cause itchiness, reddening, and swelling at contact points in subjects. To overcome these disadvantages, stiff active dry electrodes (nonintrusive) [13–15] incorporate a buffering circuit for impedance transformation and signal conditioning. Stiff (nonintrusive) passive dry electrodes doesn't require a buffering circuit, but need the conductive gel to fix them on the skin to reduce motion artifacts [16].

In recent years, considerable attention has been given to various dry electrode designs, such as Micro-Electro-Mechanical Systems (MEMS) dry electrodes (intrusive) [7,17–22], soft dry electrodes (nonintrusive) [23], dry electrophysiology electrodes using carbon nanotube (CNT) arrays (nonintrusive) [24], conductive textile-based electrodes (nonintrusive) [25], and flexible polymeric dry electrodes (nonintrusive) [26–28]. To conclude, these studies explain why dry electrodes are important for bio-potential acquisition devices.

The acquisition electrodes used affect the quality of the biomedical signal. Some dry electrodes are made of stiff substrates that can damage skin tissue when the electrode is removed [7,17–22]. Recently, Gruetzmann *et al.* [23] propose a soft and flexible electrode to improve the motion artifacts in order to reduced contact impedance for ECG measurement. Under slight pressure, the soft and flexible dry electrode on skin that has well contact with skin than stiff electrodes. Baek *et al.* were the first to propose a PDMS-based flexible dry electrode [26] for ECG measurement and PDMS is a bio-compatible material [26–29]. Kim *et al.* [27] and Moon *et al.* [28] used the same material to fabricate sensor substrates. However, connecting the wire to the PDMS electrode is challenging. Generally the conductive glue is used as the connective interface between the wire and the PDMS [26–28], which provides a weak interface connection and unstable data transmission because the conductive glue is easily detached from the PDMS-based electrode during dynamic recording sessions such as moving or running. This study proposes to transform a commercial bio-potential electrode with a conductive snap (Medi-Trace™, COVIDIEN, Mansfield, MA, USA) into an FPDE using a CO_2_ laser and replica method technology. It does not damage skin and provides a convenient wire connection and stable data transmission for long-term use.

**Figure 1.**
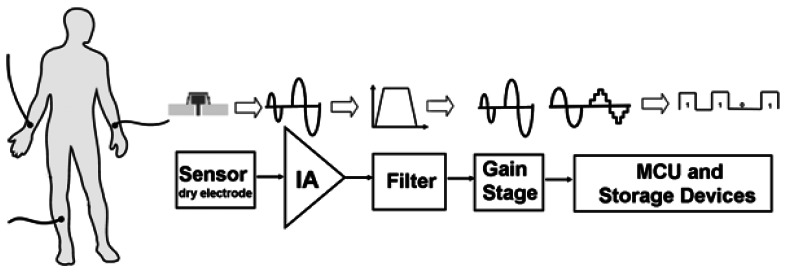
Block diagram of the portable recording device.

The design proposed in this study focuses on both the comfort and performance of bio-potential acquisition systems in order to increase the autonomy of patients and improve their quality of life. Figure 1 depicts schematically the proposed bio-potential acquisition system, which includes an FPDE and a low-power recording circuit for portable ubiquitous healthcare applications. All low-power recording circuits include state-of-the-art low-power micro-controllers, a low-power front-end acquisition chip fabricated using the standard CMOS process, and an SD card for portable use.

The front-end bio-potential acquisition chip included an ultra-low-power instrumentation amplifier (IA), filter, and gain stage. Also, noises interfere with bio-potential signals coupled to the human body. Under these circumstances, front-end circuits with a high common mode rejection ratio (C.M.R.R.), power supply rejection ratio (P.S.R.R), low-noise, and filters are required to extract signals. The proposed processing chip has low-power consumption, low noise, and high C.M.R.R. properties. These features make it a feasible bio-potential signal acquisition device. Furthermore, this proposed recording device can process and store bio-potential signal data. It is reusable, has low power consumption, and is portable. Users may record their bio-potential signals anywhere without the use of wireless receiver devices. This device can also easily be integrated with consumer electronics devices.

The FPDE integrates the low-power front-end bio-potential acquisition circuit, MCU, and SD card for the purpose of recording the ECG signal. The proposed acquisition system can be used long-term and is more comfortable than other alternatives.

## 2. Materials and Methods

### 2.1. Flexible PDMS Dry Electrode (FPDE)

**Figure 2.**
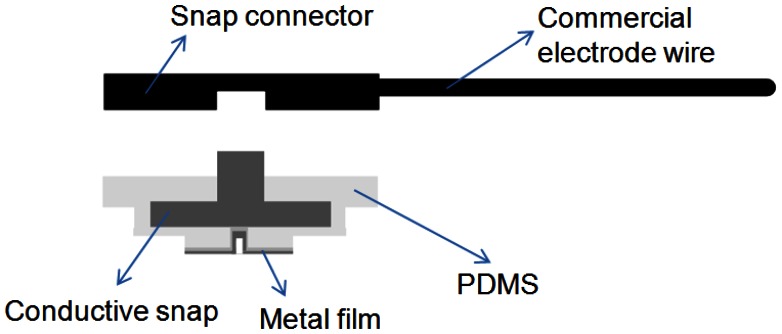
The wire connection method of FPDE.

In recent years, most laboratories have used MEMS technology to fabricate dry electrodes. For example, Baek *et al.* manufactured flexible polymeric dry electrode made of a PDMS material to measure ECG signals [26]. Their research proposed a bio-compatible material to solve the problems of using wet electrodes and MEMS dry electrodes that would cause itchiness, irritation, or skin tissue injury during long-term use. Typically conductive glue has been used as the connective interface between the wire and the PDMS [26–28]. This method provides a weak wire connection and unstable data transmission when the conductive glue is separated from the PDMS-based electrode. The proposed FPDE adopted a snap coonector instead of the glue to secure the interface connection firmly between the electrode and wire, as shown in Figure 2. The data transmission method is the same as a wet electrode that provides reliable attached for measurement, and is better than other references [26–28] for dynamic recording. The FPDE can provide stable signal transmission and can be combined with conventional hospital ECG measurement instruments.

In this study, a stair-shape polymethylmethacrylate (PMMA) master was used to fabricate the FPDE with a commercial bio-potential electrode equipped with a conductive snap. The FPDE acquired the ECG signal and transmitted it to the front-end acquisition circuit via the commercial bio-potential electrode with a conductive snap [5,7,20]. The proposed FPDE was integrated with commercial Velcro. The Velcro was firmly bonded to the surface of the FPDE and the commercial bio-potential electrode with a conductive snap combined with a medical commercial cable line (Compumedics Limited, Melbourne, Australia) was used for transmitting ECG signals. Hence, the FPDE could acquire and transmit ECG signals from skin tissue to the proposed device. This method provides the same interface for integration with conventional hospital ECG measurement instruments and portable devices as other methods. Hence, the FPDE can directly replace a wet electrode without changing the transmission interface. The proposed FPDE has the following advantages: (1) it integrates with the commercial bio-potential electrode with a conductive snap to achieve easy measurement, and (2) it does not cause itchiness, irritation, or skin tissue injury during long-term use.

This study proposes the use of a commercial CO_2_ laser machine (M300-35w, Universal Laser System Inc., Scottsdale, AZ, USA) that can produce a minimum line width of about 100 um. The laser was used to carve the PMMA plate that was employed as a mask and master for two reasons: (1) to define the pattern of the metal film and (2) to define the FPDE structure using a replica molding method. The laser technique was used to fabricate the FPDE, and the universal manufacturing methods for electrodes are used (photolithography and metal etching processes). This method can fabricate a high aspect ratio 3D structure, and thus far, no studies have used this method to fabricate electrodes and as a mask to define a metal pattern using a CO_2_ laser, which not only reduces metal etching and environmental pollution, but also the PMMA mask and master can be reused, thus reducing production time.

### 2.2. FPDE Fabrication Process

**Figure 3.**
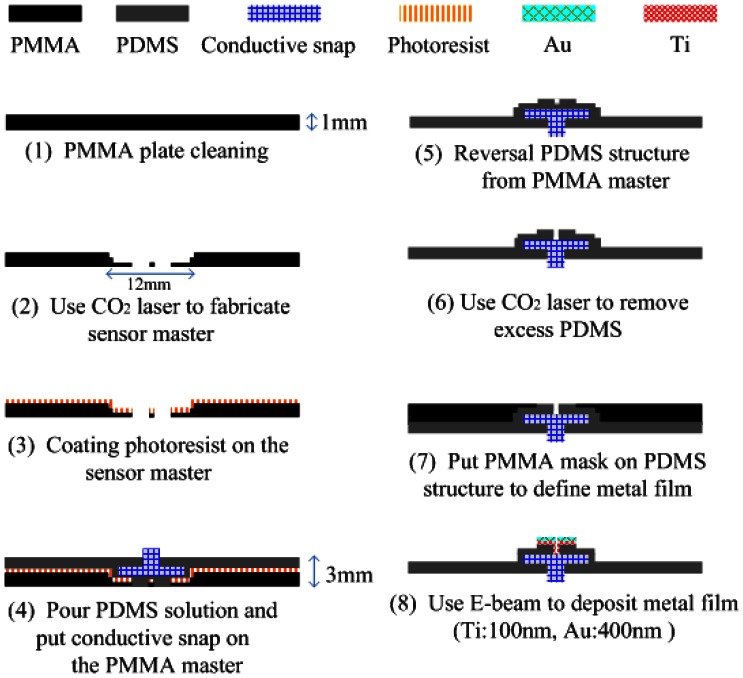
PDMS molding process flow.

The FPDE structure was fabricated using a 6 × 3 cm^2^ PMMA plate. First, the PMMA plate was cleaned with deionized (DI) water and alcohol. After cleaning, the cross graphics were defined on the sides using a CO_2_ laser. Then the AZ5214 (AZ5214, Clariant, Tokyo, Japan) was spin-coated on the master. The resist layer like a sacrificial layer can be released smoothly by acetone to protect the FPDE structure. The PDMS replica molding process is shown in Figure 3.

The PDMS solution was prepared from a mixture of Sylgard 184 silicon elastomer base and silicon elastomer curing agent (from Dow Corning, Midland, MI, USA) with a weight ratio of 10:1. After mixing, the PDMS was placed into a vacuum chamber at 750 mmHg for 30 minutes to allow trapped air bubbles produced during the mixing process to escape from the mixture. The PDMS solution was poured into the fabricated PMMA master. The PDMS was trapped in the PMMA master and cured at 70 °C for 3 hours. After curing for 3 hours, the PDMS structure was peeled off the PMMA master. The PDMS surface was exposed to an oxygen plasma ion to adhere it to the metal film [30]. Hoever, the Au surface of FPDE could flake off slightly after long-term use without interfering with ECG measurements. This work applied an O_2_ plasma to reduce flake off phenomena. The contact of the PDMS was carved by the CO_2_ laser, and Ti (100 nm) and Au (400 nm) films were evaporated using an E-beam evaporator. The deposition conditions of the metal layers are summarized in Table 1.

The FPDE metal patterns were implemented according to the following process: (1) the PDMS solution was poured, and the commercial bio-potential electrode with a conductive snap was put onto the PMMA master, (2) the PMMA mask and PDMS were placed in an e-beam chamber to deposit titanium (used as an adhesion layer). Finally, a gold pattern could be located in the center.

### 2.3. Implementation of the Acquisition Chip—DDA

**Figure 4.**
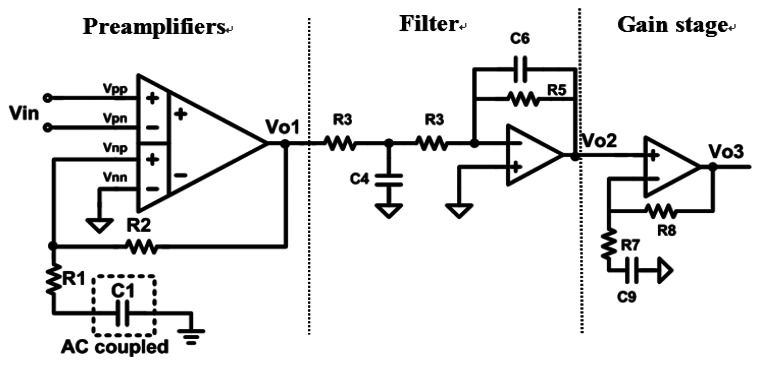
The acquisition chip.

Figure 4 schematically depicts the proposed front-end bio-potential acquisition circuits, which include three parts: preamplifiers, filters, and a gain stage.

In this study, the filter and the gain stage amplifiers were normal two-stage amplifiers. The gain of the amplifier could be tuned for various biomedical measurements by trimming the external resistor value, and the bandwidth of the filters could be designed to suit the system.

The preamplifier adopted DDA architecture for achieving high C.M.R.R., high input impedance, low-power consumption, and low-noise performance. These features make it suitable for the proposed bio-potential acquisition system application. The advantage of DDA is its simple structure, which reduces the amount of power it requires. The preamplifier adopted DDA architecture for achieving high C.M.R.R., high input impedance, low-power consumption and low noise performance. These features make it suitable for the proposed bio-potential acquisition device application. As shown in Figure 4, the resistors, R1 and R2 only affect the amplification. The DDA transfer function is presented in Equation (1) [31], which reveals that the non-inverting DDA provides unity gain in dc level input signal, and therefore it could suppress the DC biomedical offset signal without a high pass filter:

(1) $$\frac{V_{\text{out}}(j\omega )}{V_{in}(j\omega )}=\left(\frac{j\omega R_{2}C_{1}}{j\omega R_{1}C_{1}+1}\right)+1$$

**Figure 5.**
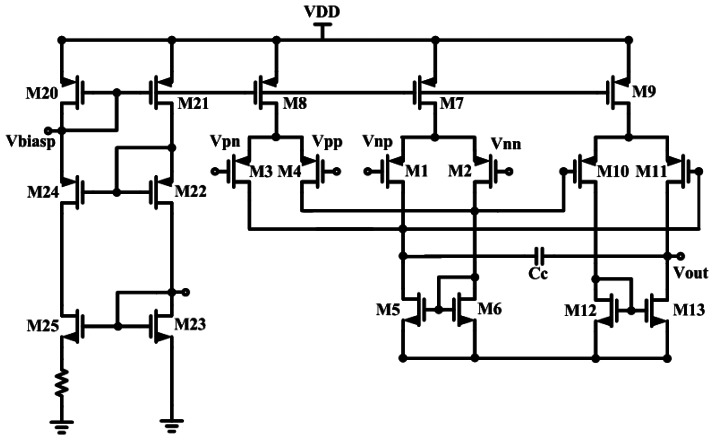
The proposed simplified DDA.

The DDA of the reference paper [20,31] adopted a three-stage structure. In this study, a simple two-stage DDA architecture is presented for obtaining a good balance between power and gain. The DDA circuit is depicted in Figure 5. However, both the left and right arms of users were connected to the V_pp_ and V_pn_ of the DDA, and the legs were connected with a V_nn_ node for the purpose of measuring ECG signals.

In addition to the simple structure, the bias current of the DDA circuit can be adjusted and reduced as much as possible in order to decrease power consumption, but the open loop gain of the DDA circuit must be sufficient to meet the specification at the same time. In order to reduce the original flicker noise, the input pair transistors (M1-M2 and M3-M4) are longer than those in the PMOS design. The second-stage amplifier is not as important for noise performance. Therefore, the input and load devices are sized to assist common-centroid layout and matching. Miller capacitance Cc is used to increase the phase margin. Table 2 shows the performance of the DDA circuit.

### 2.4. Peripheral Devices for Data Transmission and Storage

An ultra-low-power high-performance 16-bit RISC microcontroller (MSP430F149, Texas Instruments, Dallas, TX, USA) and an SD card were selected [35]. The microcontroller consumes 0.62 mW in active mode and one channel is used to record ECG signal, but the adopted MCU can offer 8-channel (max.). Its low standby power (3.6 μW), and fast wakeup time enable duty-cycle operation of the microcontroller. The microcontroller can process biomedical analog signals using the built-in ADC, and the resolution on ADC is sufficient for ECG/electroencephalogram (EEG) acquisition. The low-power dissipation feature is very suitable for long-term monitoring. The MSP430F149 links to a storage device (SD card) and provides communication data. In this system, the microcontroller performs the functions of processing bio-potential signals, power management, and controlling SD card communication. The SD card, a standard flash memory card can be used for portable application [36] due to small size and low power consumption. Recording for 24 hours requires about 85MB for 2bytes per sampling with 512Hz sampling rate as Equation (2), and a SD card with 1 GB capacity allows data storage for more than one week:

(2) 1channel×2bytes×512Hz×24houes×minutes×60seconds=84.375Mbytes

## 3. Results and Discussion

### 3.1. The Package FPDE

**Figure 6.**
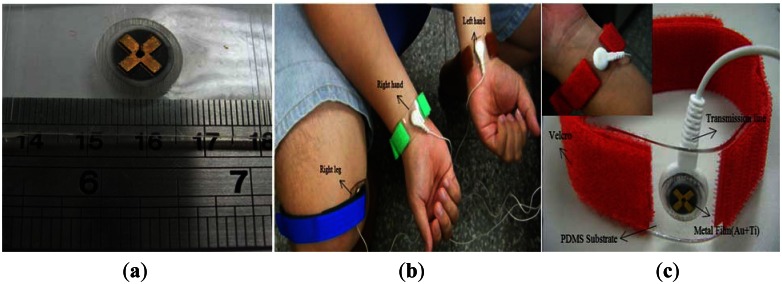
(**a**) Picture of fabricated electrode (**b**) The FPDE placed on body for measurements (**c**) Package of FPDE.

In this study, an FPDE was successfully fabricated. The Ti (100 nm) and Au (400 nm) layers were deposited stably on the surface of a PDMS structure, and metal patterns were created effectively by using a PMMA plate as a mask, which was illustrated in Figure 6(a). The electrode layer was uniformly deposited on the convex shape structure, and the contact area was about 27.3 mm^2^. To make the FPDE convenient for measuring ECG signals, the FPDE was placed on a subject's body for measurement and integrated with Velcro used as an adhesive, as shown in Figure 6(b and c).

The Velcro has viscous glue that was firmly bonded to the surface of the FPDE, and a commercial bio-potential electrode with a conductive snap was used via a connection wire intended to transform ECG signals from skin tissue to the acquisition device. This provides a convenient and stable method for combination with conventional hospital ECG measurement instruments or portable devices.

### 3.2. Measuring Impedance of FPDE

**Figure 7.**
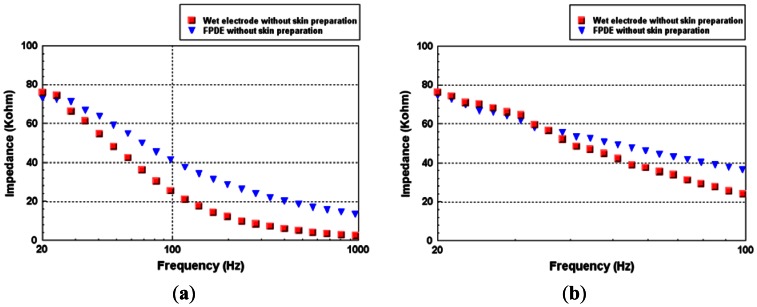
Impedance of the FPDE (contact area:27.3 mm^2^) and wet electrodes (contact area:314.2 mm^2^) at (**a**) 20 to 1 kHz and (**b**) 20 to 100 Hz.

The impedances *versus* frequency of the FPDE and wet electrodes at (a) 20 to 1 KHz and (b) 20 to 100 Hz are shown in Figure 7. For measuring both the impedances of the FPDE and the wet electrode, a precision LCR meter (Agilent E4980A precision LCR meter, Agilent Technologies, Inc., Santa Clara, CA, USA) was adopted. The LCR meter can support impedance measurement of two terminal components, such as capacitors, inductors, and resistors components [37]. Hence it was very suitable for measuring the impedances of the electrodes. The wet electrode and the proposed FPDE were both placed on the forearm. The measurement positions of the electrodes were set in the center of the anterior surface (between the wrist and the elbow) of the user's arm. The impedances were recorded between each pair of electrodes according to the change in frequency (20–1 kHz and 20–100 Hz) with a signal level of 5 mV. Twenty tests were performed on five different subjects in this study, and the impedance value of the FPDE was similar to the wet electrode during the biomedical frequency band. The change trend of the impedance was also similar to that of Karilainen's and Baek's results [38,26].

**Figure 8.**
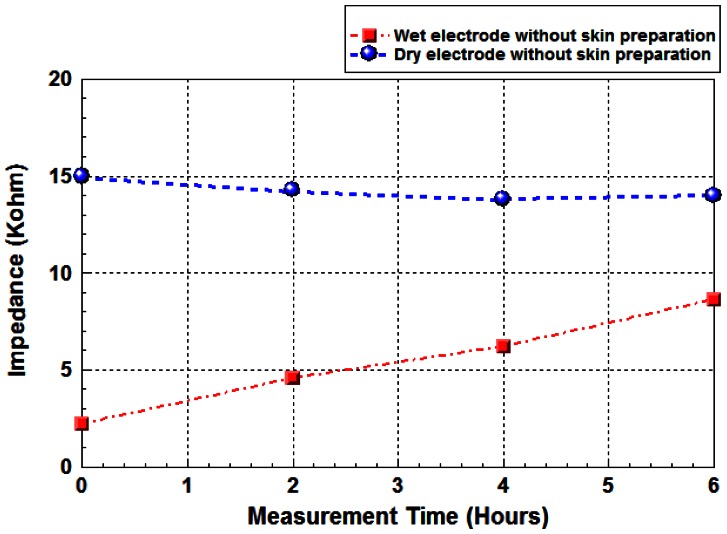
Impedance variations of FPDE and wet electrodes for long-term test.

Figure 8 shows the significant impedance variations of wet electrodes with long-term test (6 hours) at 1 kHz for five subjects, but not for the proposed FPDE electrode. This is because the conductive gel is getting dry during long-term use [26].

### 3.3. Bio-Potential Acquisition System

**Figure 9.**
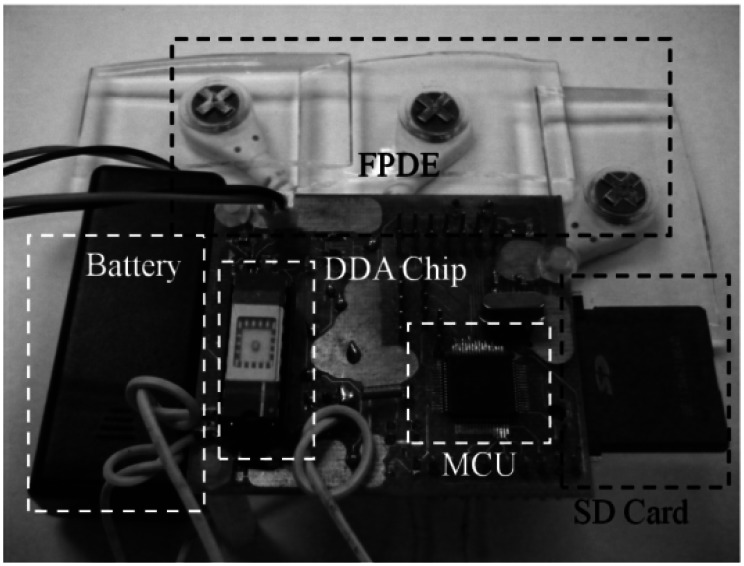
Picture of the proposed recording device.

This proposed acquisition system was implemented and integrated with novel FPDE sensors, low-power bio-potential acquisition chip, MCU, and an SD card. Table 3 summarizes the performance of the proposed acquisition device. The power consumption value of this work is better than the Holter ECG system [39]. In addition to these systems, Holter recorder [40], Digitrak XT [41] and Zio patch (iRhythm) [42] obtain more excellent power performance than this work. Nevertheless, this work provides not only novel FPDE but also low-power bio-potential acquisition chip for ECG signal acquisition. The acquisition chip consumes only 0.01% of total power consumption, while the commercial component (SD card, regulator and MCU) requires 99.9% power consumption for system integration. Especially, the commercial component SD card is not considered for low power consumption, it consumes most of power. In future, the power consumption, size and weight of the system may be significantly improved by SOC integration of all components. This will also allow the memory storage power consumption to no longer dominate. The resulting system would significantly outperform the devices listed in Table 4 in terms of size, weight and power consumption. Furthermore, the proposed device could be added data compression algorithm to save power for more individualized health care application in the future. Figure 9 shows the proposed device, including FPDE and the low-power bio-potential recording device.

**Figure 10.**
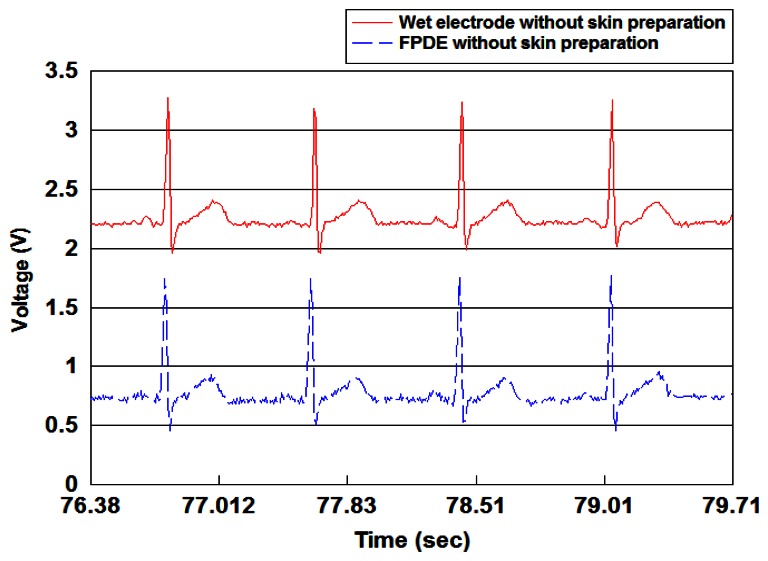
ECG signals detected by the proposed FPDE and wet electrodes.

The size of the device PCB was 5.8 × 5.0 cm^2^, and the device could be powered by two AA batteries used as the power supply. Figure 10 indicates the proposed FPDE is able to record ECG signals as clearly as the general wet electrodes.

## 4. Conclusions

In this paper, a commercial bio-potential electrode with a conductive snap was successfully adapted to a proposed FPDE that could provide reliable and robust attachment for a measurement method using a CO_2_ laser and replica method technology. It increases sensing stability and comfort for the purpose of long-term use. Additionally, the structure of the FPDE can be successfully fabricated using the replica method, and the mask made by the CO_2_ laser can be used to define metal film with a PMMA plate. Hence, the FPDE fabrication process proposed in this work provides a simple and stability deposition method to define metal pattern on a PDMS substrate, and the FPDE can obtain ECG signals efficiently and comfortably. Furthermore, we also successfully propose an ultra-low-power bio-potential acquisition chip and a storage device using an SD card for portable ECG recording. This bio-potential acquisition system not only makes the sensor more comfortable to wear, but also reduces the power consumption of portable applications. The measurement results show that the low-power bio-potential acquisition system could be applied to individualized healthcare services or integrated with a smart sensing system for normal homecare applications.

## Figures and Tables

**Table 1.** Deposition conditions of metal layers (Ti and Au) on the PDMS surface.

**Parameter**	**Conditions**
Vacuum	3×10 ^− 6^ torr
Deposition rate of Ti	0.08 – 0.12 nm/s
Thickness of Ti	100 nm
Deposition rate of Au	0.08 – 0.12 nm/s
Thickness of Au	400 nm
E-beam focusing size	3 mm
E-beam power	7 kV

**Table 2.** Performance summary of the DDA circuit.

	**This Work**	**JSSC 2008** [32]	**JSSC 2003** [33]	**TCAS 1998** [34]
Type	DDA	CBIA	OTA	CBIA
C.M.R.R. (dB)	102	120	86	99
P.S.R.R. (dB)	60	80	85	40
POWER (μW)	12.28	60	80	4,500
Input referred noise (μVrms)	0.36 (0.3–100 Hz)	0.59 (0.5–100 Hz)	2.2 (0.5–50 kHz)	1.4 (0.3–150 Hz)
Impedance (Ω)	>1 G	>1 G	none	none
Core area (mm^2^)	0.509	2	0.16	none
Supply (V)	1.8	3	±2.5	9

**Table 3.** Power consumption of the proposed acquisition device.

	**The Processing Chip**	**MCU**	**SD Card**	**Regulator**
**Power Dissipation (mW)**	0.01228	0.62	83.278	0.92
**Power Dissipation (%)**	0.01	0.73	98.17	1.1
**Total Power**	84.83 mW			
**Device Lifetime**	3 days (Two 2,500mAh AA batteries)			
**Storage Value**	One channel ECG data 85 MB/day			
**Size**	5.8 × 5.0 cm^2^			

**Table 4.** Comparison between our system and other recent similar works.

	**This Work**	**IEEE EMBS 2006** [9]	**Holter ECG System** [39]	**Holter Recorder** [40]	**Digitrak XT 2008** [41]
**Channel**	1 (max. 8-channel)	3	12	12	3
**Size**	5.8 × 5.0 × 0.4 cm^3^	N/A	N/A	8.8 × 5.5 × 2.1 cm^3^	9.1 × 5.5 × 1.9 cm^3^
**Supply (V)**	3	3	3	1.5	1.5
**Power (mW)**	84.83	375	312.5	25	10.7
**Storage medium**	SD card	SD card	Build-in storage memory	Build-in storage memory	Build-in storage memory
**Weight (g)**	38 (exclude battery)	N/A	N/A	100	62 (exclude battery)
**Electrode**	FPDE	Commercial electrode	Commercial electrode	Commercial electrode	Commercial electrode
